# An Integrated Decision-Making Approach Based on q-Rung Orthopair Fuzzy Sets in Service Industry

**DOI:** 10.1007/s44196-022-00069-6

**Published:** 2022-02-24

**Authors:** Yeter Demir Uslu, Hasan Dinçer, Serhat Yüksel, Erman Gedikli, Emre Yılmaz

**Affiliations:** 1 School of Health, Istanbul Medipol University, Kavacık Campus, Beykoz, Istanbul, 34810 Turkey; 2 School of Business, Istanbul Medipol University, Kavacık Campus, Beykoz, Istanbul, 34810 Turkey

**Keywords:** Fuzzy logic, q-Rung orthopair fuzzy sets, Intuitionistic fuzzy sets, Strategic priorities

## Abstract

This study defines key issues for sustainable healthcare policy in COVID-19 period. For this purpose, 9 different criteria that affect vaccine hesitancy are selected with the help of a detailed literature evaluation. A novel hybrid fuzzy decision-making model is developed using DEMATEL and TOPSIS based on q-Rung orthopair fuzzy sets. A comparative evaluation has also been performed using IF DEMATEL and PF DEMATEL. The results of all different methods are almost the same that indicates the reliability and coherency of the proposed model. The findings demonstrate that religion is the most critical factor that causes vaccine hesitancy. It is also defined that active population in daily life is the most important alternative. Developing countries should mainly focus on the actions regarding the religious issues to have sustainable healthcare policies in COVID-19 period. In this context, religious leaders can be released to the media and give information that the vaccine is not against religious rules. This has a significant contribution to convince people who are against the vaccine. Furthermore, these countries should also give priorities to the active population in daily life. Because this group supports the workforce in the country very seriously, it can be possible to increase the workforce in the country by completing the vaccination of this group that helps to boost economic development.

## Introduction

Finding vaccines against the COVID-19 virus has been a hope for countries. Countries that have serious problems both in the health sector and in the economy have sought ways to procure the necessary amount of vaccine to prevent these problems [1]. However, the problem of anti-vaccination has emerged in some parts of the countries. It is possible to talk about many reasons for this problem. For instance, some people think that vaccines may have side effects [2]. Moreover, not knowing the effectiveness of the vaccines is another significant problem with respect to the vaccine hesitancy. Furthermore, because some people think that vaccines are not religiously appropriate, this problem has increased [3]. Therefore, some precautions should be taken for these countries to overcome vaccine hesitancy problem [4]. A vaccination campaign with role models in the country may be helpful. For example, statements by politicians, religious leaders, and movie actors can encourage people to get vaccinated. In addition, some restrictions may be imposed within the country for those who are not vaccinated [5].

The problem of anti-vaccination needs to be solved quickly because this pandemic caused vital damage to countries both in terms of health and economy. However, to solve this problem effectively, it is important to first determine the exact cause of the problem [6]. In this way, it will be possible to develop point-and-shoot solution suggestions. Otherwise, the pandemic will last for a long time, and this will cause other economic and social problems to increase. In this context, comprehensive analyzes for specific country groups are required. Therefore, the methodology to be used in the analyzes to be made is also very important. Thanks to MCDM techniques, it can be possible to find the most effective ones among different factors [7]. Therefore, this method will be very helpful in identifying the main causes of vaccine opposition [8].

These techniques are very popular in the literature. Decision-making trial and evaluation laboratory (DEMATEL) is considered in computing the weights of the items. This method has some advantages compared to its counterparts. For example, the DEMATEL method does not only find the weights, but also reveals the causal relationship between these criteria [9]. This makes it easier to identify key factors [10]. Similarly, technique for order preference by similarity to ideal solution (TOPSIS) does not use only the distance of the alternatives to the best result in the calculation process [11]. In addition, the distance to the worst result is used to rank among the alternatives. This is the greatest advantage of the TOPSIS method [12].

MCDM techniques are also used with fuzzy numbers, as the decision-making processes become increasingly difficult. For example, it is aimed to reach more effective decisions with the analyzes made with triangular and trapezoidal numbers. However, MCDM methods have also started to be used with newer and improved fuzzy sets in the literature. For example, membership and non-membership parameters are also used in analyses with Intuitionistic fuzzy (IF) [13, 14] and Pythagorean fuzzy (PF) sets [15, 16]. In this way, it is aimed that the results obtained will be more accurate. On the other hand, a wider scope can be included in the examination with the q-Rung orthopair fuzzy sets (q-ROFs) [17]. Thanks to this situation, it can be easier to deal with uncertainty and vagueness problems [18, 19].

Key strategies are aimed to be determined to handle COVID-19 vaccine hesitancy problem in developing economies. Within this framework, 9 different criteria that affect vaccine hesitancy are defined based on a detailed literature review. Secondly, the population-based strategic priorities for vaccine hesitancy in the COVID-19 period are ranked. For this purpose, three different population alternatives are defined that are older persons and high health risk groups, active population in daily life and adolescents. A new model is created by considering DEMATEL and TOPSIS based on q-ROFs. A comparative evaluation has also been performed using DEMATEL, IF DEMATEL and PF DEMATETM.

The contribution of this manuscript is providing specific strategies to overcome vaccine hesitancy problems in developing economies. Hence, the analysis results can lead policy makers in these countries to generate appropriate policies. With the help of these strategies, it is aimed to overcome COVID-19 problem much easily. In addition to this issue, the proposed model has also some advantages. In this model, a hybrid analysis is preferred by using both DEMATEL and TOPSIS. Thus, criteria weights are not assumed equal or not defined by the authors [20] that has a powerful contribution to the objectivity [21]. Furthermore, owing to the comparative evaluation with IF and PF sets, it becomes possible to test, validity, coherency, and reliability of the model.

Part two includes literature examination. Methodology is explained in part three. Part four gives information about the analysis results. Part five explains conclusion and discussion.

## Literature Review

There is an extensive literature on vaccine opposition. In most of these studies, the reasons for vaccine opposition were analyzed. Some researchers have emphasized that religious factors are important in the opposition to vaccination. In this context, some people think that vaccinations are not in accordance with religious rules. This is a vital barrier to increasing vaccination coverage [3]. Because these people refuse to be vaccinated because of religious rules, they will not be vaccinated even if they think it is helpful [6]. In other words, emphasizing the positive aspects of the vaccine will not be enough to persuade these people to get vaccinated [22]. Reference [23] reached a conclusion that religious factors may have an important influence on minimization of this problem. Reference [24] also stated that religious leaders play a critical role to reduce COVID-19 vaccine hesitancy.

People experience anxiety about the side effects that leads to opposition to vaccines. A significant number of people fear that they will cause other diseases when they are vaccinated [25]. Although people with anxiety think that vaccines are beneficial, they do not prefer to be vaccinated [26]. The most important way to persuade these people is to share information clearly [27]. Especially with the help of statistical information, unrealistic information about the side effects of vaccines should be prevented. Reference [28] tried to identify key issues that cause vaccine hesitancy in COVID-19 period. They discussed that because people are afraid of the vaccines, they refuse to get vaccinated. Reference [29] also identified that to overcome fear problem that leads to vaccine hesitancy, statistical information should be shared so that it becomes much easier to persuade these people.

Mutation of viruses is another factor that causes anti-vaccine opposition. Especially as seen in the COVID-19 pandemic, the virus mutates several times. In addition, there are some concerns that the mutant virus will be more dangerous [30]. Because of all these problems, some people may refuse to be vaccinated. The reason for this is that people think that vaccines cannot prevent mutated viruses [31]. To prevent such problems, especially scientists should make some explanations that vaccines are also effective on mutated viruses [32]. This may be more effective on these people. Ref. [33] examined the critical factors that lead to vaccine hesitancy in COVID-19 period. They highlighted that mutation of the virus has an important effect to increase vaccine hesitancy problem.

The thought that diseases are not very effective is another factor that leads to opposition to vaccines. This problem has been encountered very frequently, especially in the COVID-19 pandemic [34]. This virus, which has a very low death rate, did not disturb some people. These people, who believe that even if the virus is approached, can be easily overcome, they find it unnecessary to be vaccinated [35]. Changing these approaches of people is important to prevent dangerous epidemic diseases such as COVID-19 [36]. In this context, short video recordings explaining the dangers of this epidemic in detail should be shared with the public. Reference [37] tried to examine vaccine hesitancy problem in United States. They concluded that some people oppose vaccines because they think that the virus is not so effective. Reference [38] also stated the significance of this issue.

Some important points can be identified with the literature examination. In a significant part of the studies, the factors causing anti-vaccination were examined. In this context, the impact of certain factors on anti-vaccine is generally discussed. However, a comprehensive priority analysis should be carried out to find more crucial determinants of anti-vaccination. The reasons for anti-vaccination may differ for country groups. In this context, a study in which many variables are considered will help to produce more effective strategies in the fight against this pandemic. This manuscript tries to define important strategies to overcome COVID-19 vaccine hesitancy problem in developing economies. Nine factors that affect vaccine hesitancy are selected with the help of a detailed literature review. Next, the population-based strategic priorities for vaccine hesitancy in the COVID-19 period are ranked. A new hybrid fuzzy decision-making model is created by considering DEMATEL and TOPSIS with q-ROFs. A comparative evaluation is performed by using DEMATEL, IF DEMATEL and PF DEMATEL.

## Methodology

This section includes q-ROFs, DEMATEL and TOPSIS approaches.

### q-ROFSs

Intuitionistic fuzzy sets (I) consider membership and non-membership degrees with the aim of having more effective results. Equation (1) illustrates these sets [13].

1 $$I=\left\{\langle {\vartheta ,\mu }_{I}(\vartheta ),{n}_{I}(\vartheta )\rangle /\vartheta \epsilon U\right\}.$$

In these sets, the condition of $0\le {\mu }_{I}\left(\vartheta \right)+{n}_{I}\left(\vartheta \right)\le 1$ should be met. Additionally, ${\mu }_{I}(\vartheta )$ and ${n}_{I}(\vartheta )$ indicate intervals of belongingness and non-belongingness [14].

Pythagorean fuzzy sets (*P*) also aims to handle uncertainty in a more effective way [15]. Equation (2) shows the details. Membership and non-membership parameters are shown as ${\mu }_{P}$ and ${n}_{P}$.

2 $$P=\left\{\langle {\vartheta ,\mu }_{P}(\vartheta ),{n}_{P}(\vartheta )\rangle /\vartheta \epsilon U\right\}.$$

While using these fuzzy sets, the condition in Eq. (3) should be satisfied [16].

3 $$0\le {\left({\mu }_{P}\left(\vartheta \right)\right)}^{2}+{\left({n}_{P}\left(\vartheta \right)\right)}^{2}\le 1.$$

Q-ROFSs are the extension of *I* and *P* with the aim of solving complex problems [17]. These sets are identified in Eq. (4). In this case, ${\mu }_{Q}$ and ${n}_{Q}$ demonstrates the degrees.

4 $$Q=\left\{\langle {\vartheta ,\mu }_{Q}(\vartheta ),{n}_{Q}(\vartheta )\rangle /\vartheta \epsilon U\right\}.$$

Equation (5) identifies the condition [18].

5 $$0\le {\left({\mu }_{Q}\left(\vartheta \right)\right)}^{q}+{\left({n}_{Q}\left(\vartheta \right)\right)}^{q}\le 1 , q\ge 1.$$

**Fig. 1:**
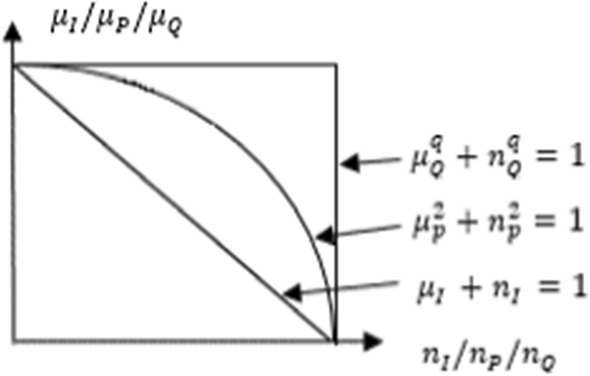
IFS, PFS, and q-ROFSs

Figure 1 compares these three different fuzzy sets.

Equation (6) indicates the degree of indeterminacy.

6 $${\pi }_{Q}\left(\vartheta \right)={\left({\left({\mu }_{Q}\left(\vartheta \right)\right)}^{q}+{\left({n}_{Q}\left(\vartheta \right)\right)}^{q}-{{\left({\mu }_{Q}\left(\vartheta \right)\right)}^{q}\left({n}_{Q}\left(\vartheta \right)\right)}^{q}\right)}^{1/q}.$$

Equations (7)–(11) include operational details [19].

7 $${Q}_{1}=\left\{\langle {\vartheta ,{Q}_{1}(\mu }_{{Q}_{1}}(\vartheta ),{n}_{{Q}_{1}}(\vartheta ))\rangle /\vartheta \epsilon U\right\} \mathrm{and} {Q}_{2}=\left\{\langle {\vartheta ,{Q}_{2}(\mu }_{{Q}_{2}}(\vartheta ),{n}_{{Q}_{2}}(\vartheta ))\rangle /\vartheta \epsilon U\right\},$$

8 $${Q}_{1}{\oplus Q}_{2}={\left({\left({\mu }_{{Q}_{1}}^{q}+{\mu }_{{Q}_{2}}^{q}-{\mu }_{{Q}_{1}}^{q}{\mu }_{{Q}_{2}}^{q}\right)}^{1/q}, {n}_{{Q}_{1}}{n}_{{Q}_{2}}\right) },$$

9 $${Q}_{1}{\otimes Q}_{2}={\left({\mu }_{{Q}_{1}}{\mu }_{{Q}_{2}}, {\left({n}_{{Q}_{1}}^{q}+{n}_{{Q}_{2}}^{q}-{n}_{{Q}_{1}}^{q}{n}_{{Q}_{2}}^{q}\right)}^{1/q}\right) },$$

10 $$\lambda Q={\left({\left(1-{\left(1-{\mu }_{Q}^{q} \right)}^{\lambda }\right)}^{1/q} , {\left({n}_{Q}\right)}^{\lambda }\right),\lambda >0 },$$

11 $${Q}^{\lambda }={\left( {\left({\mu }_{Q}\right)}^{\lambda }, {\left(1-{\left(1-{n}_{Q}^{q} \right)}^{\lambda } \right)}^{1/q}\right),\lambda >0 }.$$

Equation (12) is considered in defuzzification process.

12 $$S\left(\vartheta \right)={\left({\mu }_{Q}(\vartheta )\right)}^{q}-{\left({n}_{Q}\left(\vartheta \right)\right)}^{q}.$$

### DEMATEL

DEMATEL aims to find more crucial factors for a purpose [9]. First, direct relation matrix (RM) is obtained with Eq. (13).

13 $$A=\left[\begin{array}{cccccc}0& {a}_{12}& & & \cdots & {a}_{1n}\\ {a}_{21}& 0& & & \cdots & {a}_{2n}\\ {a}_{31}& & 0& & \cdots & {a}_{3n}\\ \vdots & \vdots & \vdots & \ddots & & \vdots \\ {a}_{n1}& {a}_{n2}& \cdots & & \cdots & 0\end{array}\right].$$

It is normalized with Eqs. (14) and (15).

14 $$B=\frac{A}{{\mathrm{max}}_{1\le i\le n}\sum_{j=1}^{n}{a}_{ij}},$$

15 $$0\le {b}_{ij}\le 1.$$

Total RM is generated by Eq. (16)

16 $$\underset{k\to \infty }{\mathrm{lim}}{\left(B+{B}^{2}+\dots +{B}^{k}\right)=B(I-B)}^{-1}.$$

The sums of rows (*D*) and columns (*E*) are defined with formulas (17) and (18).

17 $$D={\left[\sum_{j=1}^{n}{e}_{ij}\right]}_{nx1},$$

18 $$E={\left[\sum_{i=1}^{n}{e}_{ij}\right]}_{1xn}.$$

The sum of these values is used to find the weights whereas causal relationship is identified with the difference of them. Additionally, Eq. (19) is also considered in causality analysis [10].

19 $$\alpha = \frac{\sum_{i=1}^{n}\sum_{j=1}^{n}\left[{e}_{ij}\right]}{N}.$$

### TOPSIS

TOPSIS can be considered to rank alternatives [11]. First, Eq. (20) is used to find normalized values.

20 $${r}_{ij}= \frac{{X}_{ij}}{\sqrt{\sum_{i=1}^{m}{X}_{ij}^{2}}}.$$

Equation (21) is used to calculate weighted values.

21 $${v}_{ij}={w}_{ij}\times {r}_{ij}.$$

Equations (22) and (23) are considered to find positive (${A}^{+}$) and negative (${A}^{-}$) ideal solutions^12^.

22 $${A}^{+}=\left\{{v}_{1j},{v}_{2j},\dots ,{v}_{mj}\right\}=\left\{\mathrm{max}{v}_{1j} for \forall j\in n\right\},$$

23 $${A}^{-}= \left\{{v}_{1j},{v}_{2j},\dots ,{v}_{mj}\right\}=\left\{\mathrm{min}{v}_{1j} for \forall j\in n\right\}.$$

The distances to the best (${D}_{i}^{+}$) and worst solutions (${D}_{i}^{-}$) are computed by formulas (24) and (25).

24 $${D}_{i}^{+}= \sqrt{\sum_{j=1}^{n}{\left({v}_{ij}-{A}_{j}^{+}\right)}^{2}},$$

25 $${D}_{i}^{-}= \sqrt{\sum_{j=1}^{n}{\left({v}_{ij}-{A}_{j}^{-}\right)}^{2}}.$$

Equation (26) is used to compute relative closeness to the ideal solutions (${RC}_{i}$).

26 $${RC}_{i}= \frac{{D}_{i}^{-}}{{D}_{i}^{+}+{D}_{i}^{-}}.$$

## Analysis Results

**Fig. 2:**
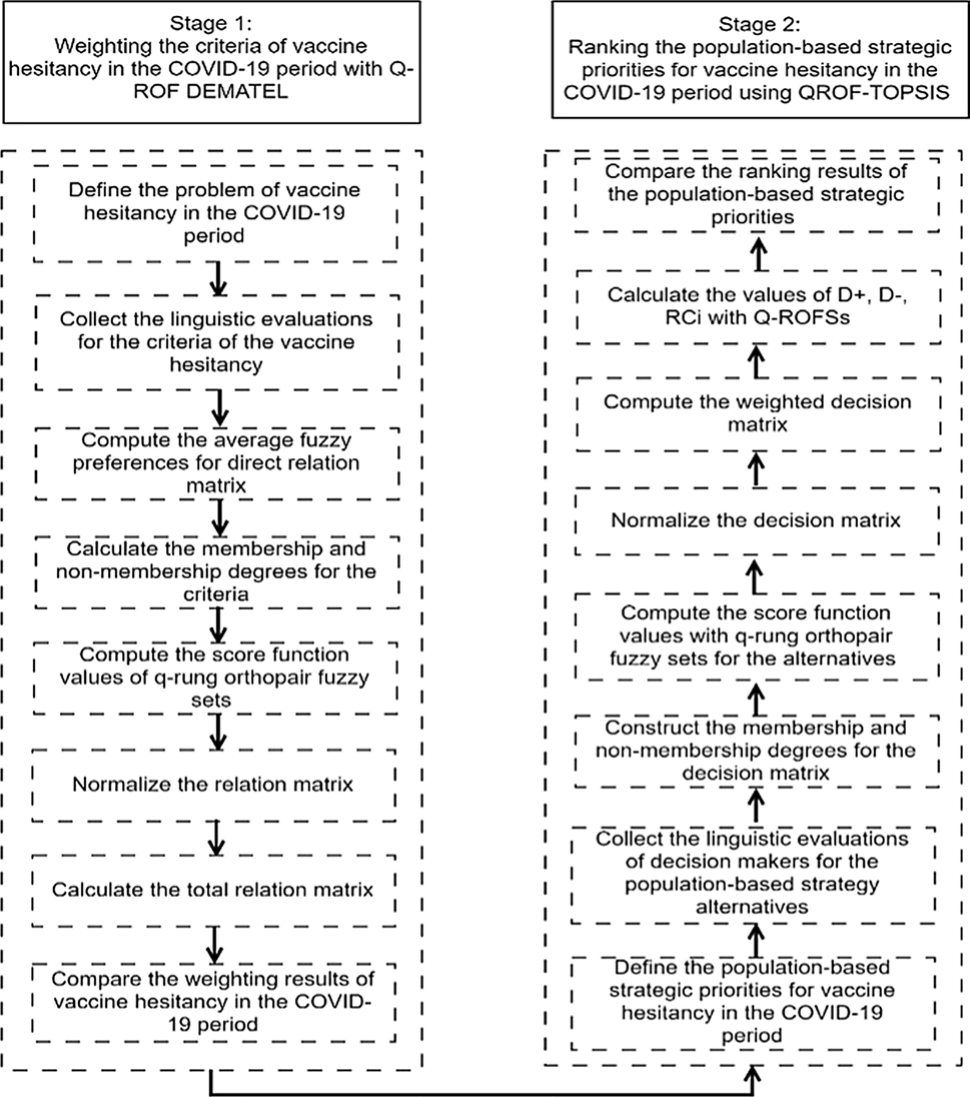
The flowchart

Appropriate strategies are aimed to be determined to cope with vaccine hesitancy problem in developing economies. A new model is generated by considering DEMATEL and TOPSIS based on q-ROFs. Figure 2 gives information about suggested model.

- Stage 1: Weighting the criteria with Q-ROF DEMATEL.
- Step 1: Defining the problem.
- Step 2: Collecting the linguistic evaluations (LEs) for the criteria of the vaccine hesitancy.
- Step 3: Computing the average fuzzy preferences for direct RM.
- Step 4: Calculating the degrees for the criteria.
- Step 5: Computing the score function values of q-ROFs.
- Step 6: Normalizing the RM.
- Step 7: Calculating the total RM.
- Step 8: Comparing the weighting results.
- Stage 2: Ranking the population-based strategic priorities.
- Step 9: Defining the population-based strategic priorities.
- Step 10: Collecting the LEs for the population-based strategy alternatives.
- Step 11: Constructing the degrees for the decision matrix.
- Step 12: Computing the score function values with q-ROFs for the alternatives.
- Step 13: Normalizing the DM.
- Step 14: Computing the weighted DM.
- Step 15: Calculating the values of *D* + , *D*–, RCi with Q-ROFSs.
- Step 16: Compare the ranking results of the population-based strategic priorities.

In the analysis process, first, criteria that affect vaccine hesitancy are identified based on a detailed literature review. Table 1 defines 9 different criteria with respect to this issue.

**Table 1:** The criteria of vaccine hesitancy in the COVID-19 period

Factors	Literature
Time (D/1)	[24]
Efficiency (D/2)	[22]
Mortality (D/3)	[28]
Origin of production (D/4)	[26]
Religion (D/5)	[31]
Information (D/6)	[34]
Personnel (D/7)	[30]
Mutation (D/8)	[25]

Time (D/1) represents not knowing the side effects. Hence, there is a positive correlation between this factor and vaccine hesitancy. Efficiency (D/2) includes not trusting the efficacy of the vaccine. Thus, it has an increasing impact on the vaccine hesitancy. Moreover, mortality (D/3) states that because of the low mortality rate of coronavirus disease, people feel hesitant for COVID vaccine. Distrust of vaccines procured from abroad explains the factor of origin of production (D/4). Furthermore, some people may think that the vaccines may not be religiously appropriate (D/5) that increases hesitancy. Information obtained from social media and other sources can also have a negative impact on this issue (D/6). The factor of personnel defines the failure to provide adequate guidance and/or information by healthcare personnel (D/7). The thought that the vaccine will not be effective due to the constant mutation of the virus can create hesitancy regarding the vaccines (D/8). Table 2 includes the scales considered in the analysis.

**Table 2:** Linguistic scales, membership, and non-membership degrees

Linguistic scales for criteria	Linguistic scales for alternatives	Membership degrees	Non-membership degrees
No influence (P)	Weakest (F)	0.10	0.90
somewhat influence (R)	Poor (G)	0.30	0.70
medium influence (S)	Fair (H)	0.60	0.40
high influence (T)	Good (I)	0.80	0.20
very high influence (U)	Best (J)	0.90	0.10

Three different experts (ETs) evaluated the items. These people have at least 17-year experience and sufficient knowledge regarding health management. Evaluations for the factors are detailed in Table 3.

**Table 3:** LEs for the factors

	D/1			D/2			D/3			D/4			D/5			D/6			D/7			D/8		
	ET1	ET2	ET3	ET1	ET2	ET3	ET1	ET2	ET3	ET1	ET2	ET3	ET1	ET2	ET3	ET1	ET2	ET3	ET1	ET2	ET3	ET1	ET2	ET3
D/1				U	U	S	T	T	T	U	S	R	T	T	U	U	T	T	T	T	T	S	S	T
D/2	S	S	R				S	R	R	T	P	P	S	R	R	U	U	S	U	U	U	T	T	S
D/3	T	R	R	U	U	U				T	R	R	U	U	U	S	S	S	S	S	S	T	T	T
D/4	T	T	T	T	T	T	T	T	T				S	S	S	U	U	U	T	R	R	U	R	R
D/5	U	T	U	U	T	T	T	T	U	S	S	S				T	T	T	T	T	T	T	T	T
D/6	T	T	R	T	T	T	U	R	R	S	S	S	T	T	T				S	S	S	S	R	T
D/7	S	T	T	S	T	T	S	S	S	T	S	S	U	T	T	T	R	R				S	R	S
D/8	S	S	S	T	R	R	T	T	T	S	S	S	T	T	T	S	S	S	T	T	T			

Average fuzzy preferences for direct RM are defined as in Table 4.

**Table 4:** Average fuzzy preferences

	D/1	D/2	D/3	D/4	D/5	D/6	D/7	D/8
D/1		0.80	0.80	0.60	0.83	0.83	0.80	0.67
D/2	0.50		0.40	0.33	0.40	0.80	0.90	0.73
D/3	0.47	0.90		0.47	0.90	0.60	0.60	0.80
D/4	0.80	0.80	0.80		0.60	0.90	0.47	0.50
D/5	0.87	0.83	0.83	0.60		0.80	0.80	0.80
D/6	0.63	0.80	0.50	0.60	0.80		0.60	0.57
D/7	0.73	0.73	0.60	0.67	0.83	0.47		0.50
D/8	0.60	0.47	0.80	0.60	0.80	0.60	0.80	

Table 5 defines the degrees for the factors.

**Table 5:** Membership and non-membership degrees for the factors

	D/1		D/2		D/3		D/4		D/5		D/6		D/7		D/8	
	*μ*	*v*	*μ*	*v*	*μ*	*v*	*μ*	*v*	*μ*	*v*	*μ*	*v*	*μ*	*v*	*μ*	*v*
D/1			0.80	0.20	0.80	0.20	0.60	0.40	0.83	0.17	0.83	0.17	0.80	0.20	0.67	0.33
D/2	0.50	0.50			0.40	0.60	0.33	0.67	0.40	0.60	0.80	0.20	0.90	0.10	0.73	0.27
D/3	0.47	0.53	0.90	0.10			0.47	0.53	0.90	0.10	0.60	0.40	0.60	0.40	0.80	0.20
D/4	0.80	0.20	0.80	0.20	0.80	0.20			0.60	0.40	0.90	0.10	0.47	0.53	0.50	0.50
D/5	0.87	0.13	0.83	0.17	0.83	0.17	0.60	0.40			0.80	0.20	0.80	0.20	0.80	0.20
D/6	0.63	0.37	0.80	0.20	0.50	0.50	0.60	0.40	0.80	0.20			0.60	0.40	0.57	0.43
D/7	0.73	0.27	0.73	0.27	0.60	0.40	0.67	0.33	0.83	0.17	0.47	0.53			0.50	0.50
D/8	0.60	0.40	0.47	0.53	0.80	0.20	0.60	0.40	0.80	0.20	0.60	0.40	0.80	0.20		

Score function values of q-ROFs are given in Table 6.

**Table 6:** Score function values of q-ROFs for the factors

	D/1	D/2	D/3	D/4	D/5	D/6	D/7	D/8
D/1	0.000	0.504	0.504	0.152	0.574	0.574	0.504	0.259
D/2	0.000	0.000	− 0.152	− 0.259	− 0.152	0.504	0.728	0.375
D/3	− 0.050	0.728	0.000	− 0.050	0.728	0.152	0.152	0.504
D/4	0.504	0.504	0.504	0.000	0.152	0.728	− 0.050	0.000
D/5	0.649	0.574	0.574	0.152	0.000	0.504	0.504	0.504
D/6	0.205	0.504	0.000	0.152	0.504	0.000	0.152	0.101
D/7	0.375	0.375	0.152	0.259	0.574	− 0.050	0.000	0.000
D/8	0.152	− 0.050	0.504	0.152	0.504	0.152	0.504	0.000

Table 7 includes normalized RM.

**Table 7:** Normalized RM

	D/1	D/2	D/3	D/4	D/5	D/6	D/7	D/8
D/1	0.000	0.146	0.146	0.044	0.166	0.166	0.146	0.075
D/2	0.000	0.000	0.000	0.000	0.000	0.146	0.210	0.108
D/3	0.000	0.210	0.000	0.000	0.210	0.044	0.044	0.146
D/4	0.146	0.146	0.146	0.000	0.044	0.210	0.000	0.000
D/5	0.187	0.166	0.166	0.044	0.000	0.146	0.146	0.146
D/6	0.059	0.146	0.000	0.044	0.146	0.000	0.044	0.029
D/7	0.108	0.108	0.044	0.075	0.166	0.000	0.000	0.000
D/8	0.044	0.000	0.146	0.044	0.146	0.044	0.146	0.000

Total RM is created as in Table 8.

**Table 8:** Total RM

	D/1	D/2	D/3	D/4	D/5	D/6	D/7	D/8
D/1	0.172	0.420	0.312	0.126	0.420	0.368	0.386	0.251
D/2	0.088	0.126	0.081	0.050	0.142	0.221	0.308	0.168
D/3	0.133	0.391	0.144	0.063	0.379	0.210	0.257	0.280
D/4	0.251	0.364	0.262	0.060	0.251	0.372	0.199	0.144
D/5	0.347	0.462	0.354	0.135	0.310	0.374	0.418	0.329
D/6	0.158	0.291	0.109	0.089	0.271	0.137	0.198	0.132
D/7	0.219	0.289	0.171	0.124	0.314	0.163	0.171	0.123
D/8	0.171	0.213	0.273	0.103	0.333	0.191	0.303	0.130

Weighting results are indicated in Table 9.

**Table 9:** Weighting Results

	q-ROF DEMATEL Results	DEMATEL Results	IF DEMATEL Results	PF DEMATEL Results
D/1	0.135	0.130	0.136	0.136
D/2	0.126	0.125	0.125	0.125
D/3	0.120	0.125	0.118	0.118
D/4	0.090	0.112	0.091	0.091
D/5	0.174	0.142	0.171	0.171
D/6	0.116	0.124	0.117	0.117
D/7	0.129	0.124	0.129	0.129
D/8	0.111	0.118	0.113	0.113

Table 9 indicates that religion (D/5) is the most critical factor that causes vaccine hesitancy. Additionally, time (D/1) and personnel (D/7) are other important items in this regard. Nonetheless, origin of production (D/4) and mutation (D/8) have the lowest weights. A comparative evaluation has also been performed by using DEMATEL, IF DEMATEL and PF DEMATEL and these results are also given in Table 9. These findings also show that all results are quite similar.

The population-based strategic priorities for vaccine hesitancy in the COVID-19 period are ranked using QROTMTOPSIS. First, the population-based strategic priorities are defined as in Table 10.

**Table 10:** Population-based strategic priorities for vaccination policy in the COVID-19 period

Population alternatives	Literature
PA1 (Older persons and high health risk groups)	[33]
PA2 (Active population in daily life)	[30]
PA3 (Adolescents)	[27]

The first population alternative (PA1) includes older persons and high health risk groups. Vaccination policy is quite important for this group to prevent the mortality and hospitalization. Second, active population in daily life (PA2) is taken into consideration to keep going the workforce and communication in socio-economic activities. The final group regarding the population-based strategic priorities is adolescents (PA3). With this group, it is aimed to decrease the infection risk and future contaminations. Evaluations with respect to these alternatives are given in Table 11.

**Table 11:** LEs for the population-based strategy alternatives

	D/1			D/2			D/3			D/4			D/5			D/6			D/7			D/8		
	ET1	ET2	ET3	ET1	ET2	ET3	ET1	ET2	ET3	ET1	ET2	ET3	ET1	ET2	ET3	ET1	ET2	ET3	ET1	ET2	ET3	ET1	ET2	ET3
PA1	H	H	H	G	J	J	I	I	I	H	H	H	G	G	H	I	I	I	I	J	J	H	H	H
PA2	H	H	H	I	I	I	H	H	H	G	G	G	I	I	I	I	I	I	H	H	H	H	H	H
PA3	G	J	J	G	G	G	I	I	I	H	H	H	G	G	H	I	I	I	H	J	J	G	G	H

Table 12 includes the degrees for the decision matrix.

**Table 12:** Degrees for the decision matrix

	D/1		D/2		D/3		D/4		D/5		D/6		D/7		D/8	
	*μ*	*v*	*μ*	*v*	*μ*	*v*	*μ*	*v*	*μ*	*v*	*μ*	*v*	*μ*	*v*	*μ*	*v*
PA1	0.60	0.40	0.70	0.30	0.80	0.20	0.60	0.40	0.40	0.60	0.80	0.20	0.87	0.13	0.60	0.40
PA2	0.60	0.40	0.80	0.20	0.60	0.40	0.30	0.70	0.80	0.20	0.80	0.20	0.60	0.40	0.60	0.40
PA3	0.70	0.30	0.30	0.70	0.80	0.20	0.60	0.40	0.40	0.60	0.80	0.20	0.80	0.20	0.40	0.60

Score function values are given in Table 13.

**Table 13:** Score function values with q-ROFs for the alternatives

	D/1	D/2	D/3	D/4	D/5	D/6	D/7	D/8
PA1	0.152	0.316	0.504	0.152	− 0.152	0.504	0.649	0.152
PA2	0.152	0.504	0.152	− 0.316	0.504	0.504	0.152	0.152
PA3	0.316	− 0.316	0.504	0.152	− 0.152	0.504	0.504	− 0.152

Table 14 includes normalized decision matrix.

**Table 14:** Normalized DM

	D/1	D/2	D/3	D/4	D/5	D/6	D/7	D/8
PA1	0.398	0.610	0.692	0.707	0.000	0.577	0.776	0.707
PA2	0.398	0.792	0.209	0.000	1.000	0.577	0.182	0.707
PA3	0.827	0.000	0.692	0.707	0.000	0.577	0.603	0.000

Weighted DM is generated as in Table 15.

**Table 15:** Weighted DM

	D/1	D/2	D/3	D/4	D/5	D/6	D/7	D/8
PA1	0.054	0.077	0.083	0.063	0.000	0.067	0.100	0.078
PA2	0.054	0.100	0.025	0.000	0.174	0.067	0.023	0.078
PA3	0.112	0.000	0.083	0.063	0.000	0.067	0.078	0.000

The values of D + , D −, RCi with Q-ROFSs are shown in Table 16.

**Table 16:** The values of D + , D–, RCi with Q-ROFSs

Alternatives	D +	D–	RCi
PA1	0.185	0.159	0.463
PA2	0.129	0.215	0.626
PA3	0.216	0.117	0.351

Finally, the ranking results of the population-based strategic priorities for the vaccine hesitancy in the COVID-19 period are compared by using PF DEMATEL and IF DEMATEL. The results are shown in Table 17.

**Table 17:** Comparative ranking results of the population-based strategic priorities for the vaccine hesitancy

Alternatives	q-ROF DEMATEL-TOPSIS	PF DEMATEL-TOPSIS	IF DEMATEL-TOPSIS
PA1	2	2	2
PA2	1	1	1
PA3	3	3	3

The ranking results of all alternatives are the same. This situation shows that proposed model in this manuscript is quite coherent. The findings demonstrated that PA2 (active population in daily life) is the most critical alternative. Secondly, PA1 (older persons and high health risk groups) also plays a key role. However, PA3 (adolescents) takes the last rank.

## Discussions and Conclusions

It is aimed to state strategies to handle COVID-19 vaccine hesitancy problem in developing economies. In this context, nine different criteria that affect vaccine hesitancy are selected. A new model is created by considering DEMATEL and TOPSIS based on q-ROFs. A comparative evaluation has also been performed by using DEMATEL, IF DEMATEL and PF DEMATEL All results are quite similar that indicates the reliability and coherency of the findings. Religion is the most critical factor that causes vaccine hesitancy. Moreover, time and personnel are other significant items related to this issue. Nevertheless, origin of production and mutation have the lowest weights. It is also concluded that active population in daily life is the most important alternative. Older persons and high health risk groups also play a key role whereas adolescents take the last rank.

The analysis results give information that to handle vaccine hesitancy in COVID-19 period for developing economies, countries should mainly focus on the religious reasons. This situation gives information that people in these countries refuse COVID vaccines because they think that these vaccines are not religiously appropriate. Therefore, it would be appropriate for countries to take specific actions to solve this problem. For example, religious leaders can be released to the media and give information that the vaccine is not against religious rules. This will seriously help convince people who are against the vaccine. References [39] and [40] also stated that religious leaders play a crucial role to manage vaccine hesitancy problem in COVID-19 period.

Additionally, it is also concluded that the countries mainly give priorities to the active population in daily life. This result shows that it would be appropriate to complete the vaccination of the young group first, rather than the elderly and children. This group supports the workforce in the country very seriously. Therefore, it will be possible to increase the workforce in the country by completing the vaccination of this group. In addition to the point, the youth group is also the segment that is most involved in the society. For this reason, it is possible to reduce the circulation rate of the virus by completing the vaccination of this group. Therefore, considering both reasons, it is important for countries to take action to increase the vaccination of this group. References [41] and [42] also highlighted that necessary strategies should be developed to minimize vaccine hesitancy for especially young people.

The main contribution of this manuscript is creating specific strategies to overcome vaccine hesitancy problems in developing economies. In spite of this issue, the main limitation of the manuscript is making evaluation for only developing countries. However, the reasons of vaccine hesitancy can also differ for other country types. A new examination can be made for developed countries. Also, analytical network process (ANP) approach can be used to find the weights. Hence, it can be possible to make comparative examination.

## Data Availability

There is no data in this study.

## Abbreviations

ANP: Analytical network process
DEMATEL: Decision-making trial and evaluation laboratory
IF: Intuitionistic fuzzy
PF: Pythagorean fuzzy
q-ROFs: q-Rung orthopair fuzzy sets
